# A case report of verapamil toxicity leading to haemodynamic compromise in obstructive hypertrophic cardiomyopathy

**DOI:** 10.1093/ehjcr/ytae596

**Published:** 2024-11-20

**Authors:** Jonathan W Gau, Florian Rader, Behram Mody, Andy Y Lee

**Affiliations:** Department of Internal Medicine, School of Medicine, University of California, Irvine, 101 City Dr S, Orange, CA 92868, USA; Department of Cardiology, Cedars-Sinai Medical Center, Smidt Heart Institute, 127 S San Vicente Blvd Pavilion Suite A3600, Los Angeles, CA 90048, USA; Department of Medicine, Division of Cardiology, University of California, Irvine, 333 City Blvd W, Orange, CA 92868, USA; Department of Medicine, Division of Cardiology, University of California, Irvine, 333 City Blvd W, Orange, CA 92868, USA

**Keywords:** Obstructive hypertrophic cardiomyopathy, Verapamil toxicity, Case report, Echocardiogram

## Abstract

**Background:**

Hypertrophic cardiomyopathy (HCM) is an inherited condition characterized by left ventricular (LV) hypertrophy unexplained by increased afterload, often with concomitant outflow tract obstruction. Verapamil is commonly used to treat symptomatic patients. However, its potential for adverse effects should be recognized.

**Case summary:**

This report provides a case of cardiogenic shock in HCM, where a patient presented with chief complaint of fatigue and shortness of breath with an acute decline in LV systolic function on echocardiogram. His symptoms were attributed to verapamil toxicity following a recent increase in dosage. He achieved rapid symptom relief after holding his home medications, in addition to conservative management.

**Discussion:**

This case report aims to elucidate the potential for multiple adverse effects involved with verapamil use in patients with obstructive HCM.

**Learning points**

- Understand the haemodynamic effects of verapamil in hypertrophic cardiomyopathy.
- Appreciate verapamil toxicity/overdose as potential trigger of low cardiac output.

## Introduction

Left ventricular outflow tract obstruction (LVOTO) in hypertrophic cardiomyopathy (HCM) is a dynamic phenomenon requiring an understanding of the underlying anatomic features and its response to physiological factors. Treatment focuses on symptom relief and begins with pharmacologic agents including beta-blockers (BBs) and calcium channel blockers (CCBs). Although verapamil has long been recognized as a therapeutic alternative for patients with HCM, its use has been limited by its potential for haemodynamic compromise in HCM. We present a unique case of verapamil toxicity in HCM resulting in a low cardiac output.

## Summary figure

**Figure:**
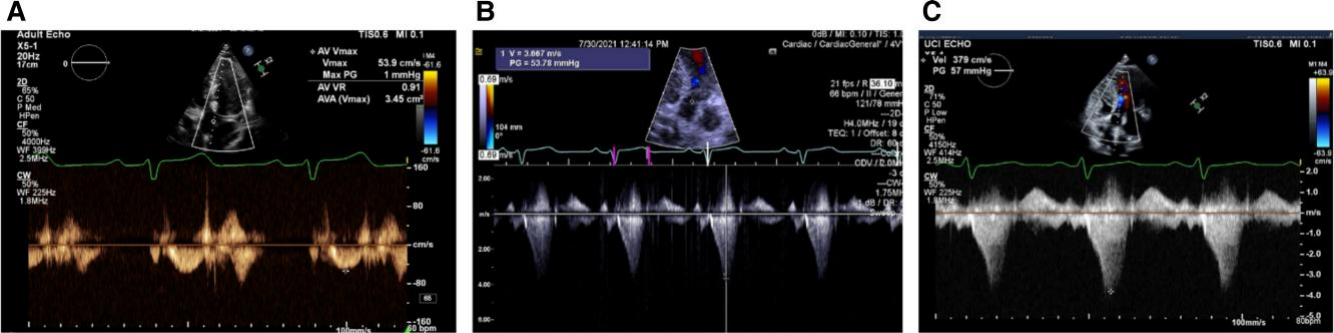
Serial echocardiograms demonstrating no left ventricular outflow gradient on admission (*A*) and dynamic peak gradients after fluid resuscitation and verapamil discontinuation on hospital Day 2 (*B*) and prior to discharge (*C*).

## Case presentation

A 34-year-old man with known history of obstructive HCM (oHCM) presented with acute fatigue, weakness, and shortness of breath. He was bradycardic to 46 b.p.m. and hypotensive to 87/55 mm Hg. His respiratory rate was 28 breaths/min, and oxygen saturation was 98% on 2 L nasal cannula. On exam, he was cool to touch, pale in appearance, and in notable respiratory distress with appreciable jugular venous distension. His physical exam was remarkable for bradycardia without systolic ejection murmurs. Lung auscultation revealed bilateral rales.

He previously underwent alcohol septal ablation at age 26 and a single chamber implantable cardioverter defibrillator (ICD) implantation for primary prevention of sudden cardiac death. He was intolerant to atenolol and disopyramide and was now taking metoprolol succinate 50 mg twice daily and a recently increased dose of verapamil 180 mg twice daily. He denied ICD shocks with no arrhythmias on ICD interrogation.

The patient’s presentation was concerning for cardiogenic shock from low cardiac output, worsened by the negative inotropic effects of his home medications. Other considerations included septic vs. obstructive shock.

**Figure 1:**
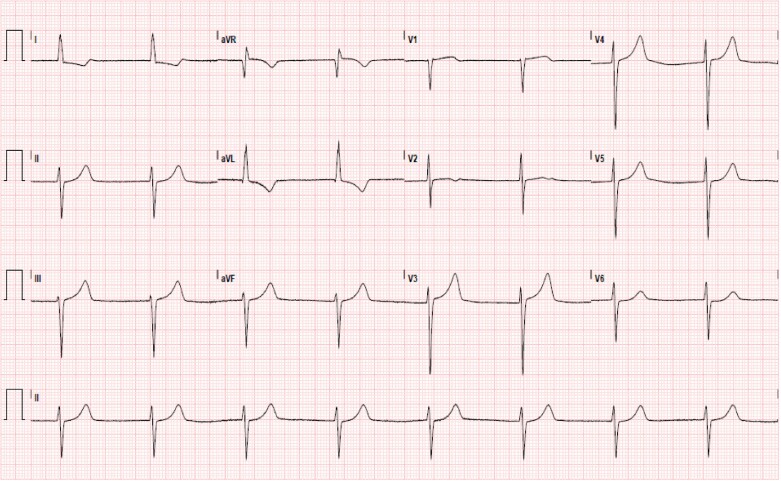
EKG. Junctional bradycardia, left axis deviation.

**Figure 2:**
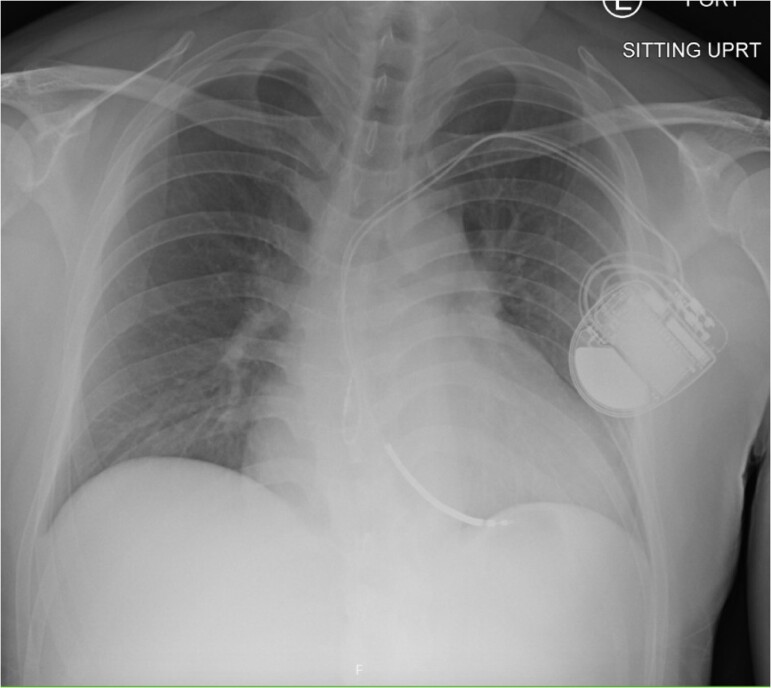
Chest X-ray. Large cardiac silhouette with vascular congestion.

His labs showed Na+ 131 mmol/L, K+ 6.7 mmol/L, HCO3− 10 mmol/L, Blood urea nitrogen 22 mg/dL, Cr 2.2 mg/dL, lactate 7.2 mmol/L, high-sensitivity troponin I 740 ng/L, and B-type natriuretic peptide 301 pg/mL. Electrocardiogram revealed junctional bradycardia, left ventricle (LV) hypertrophy, and no acute ischaemic changes (*Figure 1*). Echocardiogram revealed severe concentric LV, flattened inter-ventricular septum, LV stroke volume index of 13 mL/m² with a decrease in LV ejection fraction (LVEF) from 68% to 35%, and no LVOT obstruction (Supplementary material online, *Video S1*). Chest X-ray showed cardiomegaly and vascular congestion (*Figure 2*).

The patient’s metoprolol and verapamil were held. The atrial pacing rate on his ICD was increased to 80 b.p.m. to improve his cardiac output with a goal cardiac index of 2 L/min/m^2^. A phenylephrine infusion was initially started and then weaned off with i.v. fluids. His hyperkalaemia was treated with temporizing measures including i.v. calcium, which also led to improvement of his hypotension and bradycardia. His renal function returned to baseline by hospital Day 3. Serial echocardiograms showed a recovered ejection fraction of 75% and peak LVOT gradient of 65 mmHg (Supplementary material online, *Videos S2* and *S3*). He was restarted on metoprolol and reduced dose of verapamil 60 mg twice daily, which he tolerated well prior to discharge.

The patient’s cardiogenic shock was attributed to suspected verapamil-mediated bradycardia and profound negative inotropy exacerbated by his recent increased dose. He was closely followed in clinic with improvement in symptoms to New York Health Association Class II. Outpatient treadmill echocardiogram showed stable ejection fraction with minimal resting LV outflow tract (LVOT) gradient and peak LVOT gradient of 168 mmHg with exercise. We discussed referral to a reputable oHCM centre for septal myectomy, but per shared decision-making, the patient felt his symptoms were acceptable on medical therapy alone. Myosin inhibitors, including mavacamten, were discussed as options but deferred at the time given his recent heart failure hospitalization.

## Discussion

Our case highlights how verapamil toxicity can result in haemodynamic consequences in HCM with its inherent effects on electrophysiology, vascular resistance, and inotropy. Recognizing verapamil’s potential for adverse effects, particularly in patients with LVOTO, is important to improve clinical outcomes. Left ventricular outflow tract obstruction occurs in two-thirds of HCM cases and stems from its characteristic septal hypertrophy, displacement of the papillary muscles, and systolic anterior motion of the mitral valve leaflets often times in the setting of hyper-dynamic systolic function.^1^ The subsequent increase in LV intra-cavitary pressure worsens myocardial wall tension leading to impaired diastology. The dynamic nature of LVOTO in HCM is well described,^2^ with the degree of obstruction influenced by the load imposed on the LV. As a result, patients with oHCM are prone to symptoms on exertion and increased morbidity and mortality compared with their non-obstructive counterparts.^3^

Treatment of HCM is determined by symptom severity, phenotypic expression, and existing comorbidities.^3^ Non-dihydropyridine CCBs, specifically verapamil, are accepted therapeutic alternatives for patients BB intolerance or with refractory symptoms. The use of CCBs in HCM stems from studies suggesting that calcium-induced hyper-contractility is pathognomonic of the disease. However, contemporary evidence for their effectiveness is not available. Furthermore, verapamil’s utility is limited by its haemodynamic effects and unfavourable pharmacokinetics associated with cumulative dosing.^4^

One example of verapamil’s adverse effects in HCM is its suppression of sinoatrial and atrioventricular activity, leading to a range of effects from mild bradycardia to complete heart block.^5^ A study on i.v. verapamil in 27 patients, a majority with oHCM, found that all subjects developed PR interval prolongation.^6^ Perrot *et al.*^7^ also details a case of sudden cardiac death associated with oHCM, where the patient was noted to have episodes of paroxysmal bradycardia and later complete heart block correlating with increased verapamil doses. As with our patient, disruptions in atrial and/or ventricular conduction in oHCM can negatively affect cardiac output, resulting in a vicious cycle of hypotension and worsening obstruction.^8^

Verapamil-induced vasodilation can also potentially trigger haemodynamic compromise in HCM. Epstein *et al.*^8^ analysed the effects of hypotension in an oHCM patient receiving i.v. verapamil. He found a drop in systolic blood pressure following verapamil administration correlating with a substantial increase in LVOT gradient. The gradient immediately reversed after verapamil cessation, which was also corroborated in patients receiving oral verapamil. The mechanism is likely due to the reflex tachycardia and adrenergic vasoconstriction triggered by the vasodilatory effects of verapamil. This results in increased LV contractility and subsequently an increase in LVOT gradient.

Verapamil also exhibits negative inotropic effects. Although rarely described, excessive doses may result in reduced contractility and circulatory shock.^8^ Wilmshurst *et al*.^9^ first observed how verapamil reduced LV end-systolic pressure to volume ratio irrespective of heart rate, suggesting its negative effect on contractility. In our case, the suspected verapamil toxicity leading to depressed contractility obviated his known LVOTO, evident by lack of appreciable murmur and no LVOT gradient on echocardiogram despite his initial vasodilatory state.

While surgical septal myectomy is typically recommended in drug-refractory oHCM, percutaneous alcohol septal ablation may be a suitable alternative for those with select clinical profiles.^10^ Novel targeted therapies for oHCM including mavacamten and aficamten directly inhibit cardiac myosin and have demonstrated effectiveness in reducing LVOTO.^11^ However, these trials have specifically excluded patients with reduced LVEF and carry similar side effect profiles as potent negative inotropes.

## Lead author biography


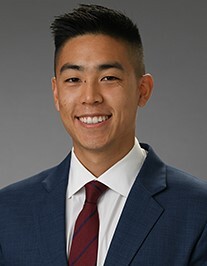


I am currently an Internal Medicine Chief Resident at the University of California, Irvine Medical Center. My interests are in cardiology, specifically Heart Failure physiology and treatments.

## Supplementary Material

ytae596_Supplementary_Data

## Data Availability

No new data were generated or analysed in the writing of this case report. The data underlying the case report, including lab work and imaging, are available in the manuscript and its Supplementary material.

## Supplementary material

**Consent:** The authors of this manuscript confirm that written patient consent has been obtained from the party involved and is currently being held by the treating institution.

**Funding:** None declared.
